# Antibiotic prophylaxis in flexible ureterorenoscopy with negative urine culture

**DOI:** 10.1002/bco2.242

**Published:** 2023-07-03

**Authors:** Daniela María Méndez‐Guerrero, Christian Buitrago‐Carrascal, Andrés Felipe Puentes‐Bernal, Dilma Alexandra Cruz‐Arévalo, Diego Camacho ‐Nieto, Marcelo Andrés Calderón, Juan Camilo Álvarez‐Restrepo, Mayra Alejandra Brijaldo‐Carvajal, Natalia Perdomo‐Bernal, María Carolina Moreno‐Matson, Milciades Ibañez‐Pinilla, José Daza‐Vergara

**Affiliations:** ^1^ Universidad del Rosario Bogotá Colombia; ^2^ Department of Urology Hospital Universitario Mayor Méderi ‐ Universidad del Rosario Bogotá Colombia; ^3^ Universidad Nacional de Colombia Bogotá Colombia; ^4^ Hospital Universitario Mayor Méderi ‐ Universidad del Rosario Bogotá Colombia

**Keywords:** antibiotics, bacteria, infections, sepsis, urolithiasis

## Abstract

**Objective:**

To improve susceptibility profiles of nosocomial bacteria, identifying the difference between infectious complications in patients undergoing endoscopic flexible ureterolithotomy (fURS) with negative urine culture (UC) that received extended antibiotic prophylaxis (EP) compared with standard antibiotic prophylaxis (SP).

**Methodology:**

This is a retrospective, observational, analytical cohort study, comparing infectious complications between patients undergoing fURS with negative UC who received EP versus SP. We include patients with susccessfull fURS, <20‐mm stones and complete information.

**Results:**

Overall, 10.3% of patients had complications, 7.2% of patients had postoperative urinary infection, 1.8% had upper urinary tract infection (UTI) and 1.4% had urinary sepsis. Lower UTI were significantly more likely in the extended prophylaxis group with 6.8% versus 2.7% (RR = 2.8; 95% CI: 1.10–7.37, *p* = 0.030). The risk of upper UTI and sepsis did not show significant differences. A total of 69% patients with postoperative infection had isolated multidrug‐resistant bacteria (MDRB) in the UC, with a higher risk in patients with extended prophylaxis (RR = 3.1; 95% CI: 1.33–7.59, *p* = 0.009).

**Conclusions:**

Patients with negative UC who underwent fURS using extended prophylaxis have two times higher risk of low UTI than patients with standard prophylaxis, without differences in the incidence of upper UTI or urinary sepsis. The risk of MDRB isolation in the postoperative UC is higher in the extended prophylaxis group, therefore we recommend the standard 60‐min preoperative prophylaxis.

## 1 INTRODUCTION

Infections after surgery lead to morbidity and increase health costs.^1^, ^2^, ^3^ The rate of infection associated with endourological procedures is reported between 1.8% and 18.3%.^4^, ^5^ With the introduction of prophylactic antibiotics, the rate of urinary infection decreases from 21% to 1–5% and also the incidence of bacteremia from 2% to 1.3%.^6^, ^7^, ^8^

The main bacteria associated with postoperative infections in urologic surgery are *Enterobacteriaceae*, *Pseudomonas aeruginosa* and *Enterococcus*.^9^ These bacteria become resistant to numerous classes of antibiotics. According to the Centers for Disease Control and Prevention (CDC) about 80% of *Enterococcus faecium* strains in Italy are resistant or intermediate resistant to aminopenicillins.^9^ *P. aeruginosa* has shown resistance to piperacillin‐tazobactam and antipseudomonal carbapenems in about 20% of urine culture (UC); likewise, resistance to fluoroquinolones is reported in 26% of cases.^9^ *Escherichia coli* is resistant to fluoroquinolones in 20–40% of cases and to third generation cephalosporins in 20% of strains.^9^

Increasing antibiotic resistance can lead to failure of therapeutic regimens and therefore it is important to assess the risk–benefit of extended prophylactic antibiotic regimens.^10^, ^11^

The EAU guideline recommended antibiotic prophylaxis to patients with infected stones, bacteriuria and in the case of internal stent placement.^1^

Best practice statements on urologic procedures establish that restricting antibiotic prophylaxis (AP) will help to minimise the risks of antimicrobial overuse.^12^ Therefore, prior to any urologic procedure, it is necessary to be taken a UC to choose the adequate AP for the procedure and in case of UTI should always receive preoperative treatment.^1^, ^12^

The guidelines affirm that there is no high‐quality evidence to defend the use of multiple doses of antimicrobials in a negative UC or a patient without presurgical symptomatic infection^12^, ^13^ and establish that a single‐dose AP is recommended before clean‐contaminated genitourinary procedures, such as intervention for ureteroscopic stone removal and all patients undergoing endourological treatment,^1^ with an antimicrobial agent chosen based on previous UC results or the local antibiogram.^12^

Also, recent authors consider single dose administration is sufficient, and there are no additional benefits to extended antibiotics treatment following ureteroscopy^12^, ^13^, ^14^ based on similar rates of postoperative UTI in patients with and without home‐going antibiotics therapy.^12^, ^13^

To improve the susceptibility profiles of nosocomial bacteria, without increasing the risk of infectious complications, we evaluated the difference between infectious complications in patients with negative UC undergoing endoscopic ureterolithotomy who were treated or not with extended AP.

## 2 MATERIALS AND METHODS

We conducted a retrospective, observational, analytical cohort study that compared infectious complications in patients with a negative UC (time period not greater than 30 days prior to the time of surgery), among the cohorts that received standard prophylaxis (60 min prior to surgery) and those who received pre‐surgical extended prophylaxis (3 days prior), between January 2013 and December 2019.

The patients included in the sample underwent endourological successful intervention with a flexible ureteroscope, and lithotripsy with Holmium: YAG laser, due to renal and ureteral lithiasis, all with negative UC (UC) and without clinical signs of infection at the time of the intervention. Patients with failed flexible endoscopic ureterolithotomy, with >20‐mm stones or without findings of stones within the urinary tract during the surgical procedure, were excluded.

For data collection, billing databases for surgical procedures were used to obtain information on patients undergoing successful flexible ureterorenoscopy (fURS) with <20‐mm stones with previous negative UC. The study was approved by the Institutional Méderi University Hospital Ethics Committee and Rosario University Ethics Committee. We included patients with multidrug‐resistant bacteria isolation and symptomatic infection in the first 30 days postoperative according to the Clavien–Dindo classification. The number of complications in the sample and the severity of the complications developed were determined according to the Clavien–Dindo classification.

Infectious complications evaluated included lower urinary tract infection (UTI), upper UTI and sepsis. These variables were compared between the two cohorts. In addition, the bacteria isolated from the postoperative UC in patients with infectious complications were described to determine the risk of developing infections by multidrug‐resistant bacteria according to the cohort.

Normality tests (Kolmogorov–Smirnov or Shapiro–Wilk depending on the size of the sample obtained) were performed. For continuous quantitative variables that are approximately normally distributed, the mean was used as a measure of central tendency and the standard deviation as a measure of dispersion. For those quantitative variables whose distribution was not normal, the median and interquartile ranges were used, respectively. Variables were recorded when necessary. Categorical variables were provided in the form of frequency tables.

A bivariate analysis of the sociodemographic variables was carried out, in addition to a multivariate analysis with the variables in which, according to their normality, a statistical significance was observed (*p* = 0.5) and were related to the dependent variables.

## 3 RESULTS

Between January 2013 and December 2019, 723 successful fURS for treatment of <20‐mm stones were performed, of which 498 had negative UC. Preoperative prophylaxis was performed in an extended manner (3 days prior to surgery) in 162 (32.5%) patients and in a standard manner (1 h prior to the procedure) in 336 (67.5%) patients. The mean age was 46.7 ± 14.5 years, 56.4% of patients were men and 66.8% of patients had preoperative urinary diversion.

Significant differences were found between the cohorts in terms of sex, stone size, preoperative hydronephrosis and preoperative diversion. In the standard prophylaxis group, more male patients underwent surgery, with 60.1% versus 48.8% in the extended prophylaxis group. The mean stone size was 13.18 mm in the extended prophylaxis group and 11.47 mm in the standard prophylaxis group. Preoperative diversion with double J‐stent and nephrostomy occurred more frequently in the standard prophylaxis group (Table 1).

**TABLE 1:** Demographic and clinical characteristics between the cohorts: extended and standard preoperative prophylaxis.

Variable	All patients (*n* = 498)	Extended prophylaxis (*n* = 162)	Standard prophylaxis (*n* = 336)	*P* value
Age (years), Mean ± SD	46.7 ± 14.5	47.3 ± 14.2	46.4 ± 14.6	0.486
Sex				0.017
Male *n* (%)	281 (56.4%)	79 (48.8%)	202 (60.1%)	
Female *n* (%)	217 (43.6%)	83 (51.2%)	134 (39.9%)	
Creatinine (mg/dL ‐ umol/L), Mean ± SD	1.09 ± 0.50	1.03 ± 0.30	1.14 ± 0.62	0.558
Glomerular filtration rate (mL/min 173 m^2^), Mean ± SD	77.53 ± 25.78	78.624 ± 25.40	76.561 ± 25.43	0.789
Stone size (millimetres), Mean ± SD	12.03 ± 5.96	13.18 ± 6.55	11.47 ± 5.58	0.006
Stone density (Hounsfield unit), Mean ± SD	1094 ± 349	1130 ± 337	1064 ± 357	0.115
Stone location *n* (%)				
Proximal ureter	192 (38.9%)	71 (44.1%)	121 (36.4%)	0.447
Renal pelvis	58 (11.8%)	22 (13.7%)	36 (10.8%)	
Lower calyx	75 (15.2%)	22 (13.7%)	53 (16.0%)	
Medium calyx	25 (5.1%)	5 (3.1%)	20 (6.0%)	
Upper calyx	16 (3.2%)	5 (3.1%)	11 (3.3%)	
Ureteral and calyx	60 (12.2%)	18 (11.2%)	42 (12.7%)	
Multiple chalices	67 (13.6%)	18 (11.2%)	49 (14.8%)	
Contralateral stone *n* (%)				
Yes	92 (18.5%)			
No	406 (81.5%)			
Previous surgical hydronephrosis				0.004
No	316 (67.5%)	80 (58.0%)	236 (71.5%)	
Grade I	61 (13.0%)	22 (15.9%)	39 (11.8%)	
Grade II	58 (12.4%)	24 (17.4%)	34 (10.3%)	
Grade III	26 (5.6%)	12 (8.7%)	14 (4.2%)	
Grade IV	7 (1.5%)	0 (0.0%)	7 (2.1%)	
Presurgical diversion				
Double J‐stent *n* (%)				
Yes	309 (62.0%)	82 (50.6%)	227 (67.6%)	<0.00
No	189 (38.0%)	80 (49.4%)	109 (32.4%)	
Nephrostomy *n* (%)				<0.001
Yes	24 (4.8%)	16 (9.9%)	8 (2.4%)	
No	474 (95.2%)	146 (90.1%)	328 (97.6%)	
Prophylactic antibiotic	440 (88.4%)	136 (84.0%)	304 (90.5%)	0.007
Cephalosporin 1st	28 (5.6%)	9 (5.6%)	19 (5.7%)	
Ciprofloxacin	3 (0.6%)	2 (1.2%)	1 (0.3%)	
Ampicillin	7 (1.4%)	1 (0.6%)	6 (1.8%)	
Amikacin	5 (1.0%)	2 (1.2%)	3 (0.9%)	
TMP/SMX	2 (0.4%)	2 (1.2%)	0 (0.0%)	
Cefepime	3 (0.6%)	3 (1.9%)	0 (0.0%)	
Gentamicin	10 (2.0%)	7 (4.3%)	3 (0.9%)	
Type of prophylaxis				
Extended	168 (32.5%)			
Standard	336 (67.5%)			

The surgical procedure was performed with a ureteral access sheath in 78.3% of patients. The access sheath was used due to surgeon's preference and because of the mean stone size, which most of them were over 10 mm and the surgeon had to make multiple trips up and down the ureter.

Postoperative urinary diversion, subject to the urologist's criteria, was performed with double J‐stent in 93.2% of patients, of which 74.5% remained with double J‐stent (with string) and was removed a week after the surgical procedure. Patients with previous diversion and nephrostomies corresponded to 0.6% and were patients who had this pre‐surgical diversion and continued in the postoperative time.

Complications occurred in 10.3% of patients, most of them minor complications, of which 7.8% were Clavien–Dindo 1 and 2. Clavien–Dindo 3a complications occurred in 0.4% of patients and corresponded to two patients who need double J‐stent removal under local anaesthesia, due to catheter‐associated symptoms and UTI. Two ureterorenoscopy (URS) were performed for residual ureteral stones, under regional anaesthesia. Mortality occurred in one patient (0.2%) in the extended prophylaxis group, due to urinary sepsis (Table 2).

**TABLE 2:** Complications according to the Clavien–Dindo classification in the general cohort (*n* = 498).

None 447 (89.7%)
1 10 (2%)
2 29 (5.8%)
3a 2 (0.4%)
3b 2 (0.4%)
4a 3 (0.6%)
4b 4 (0.8%)
5 1 (0.2%)

**FIGURE 1:**
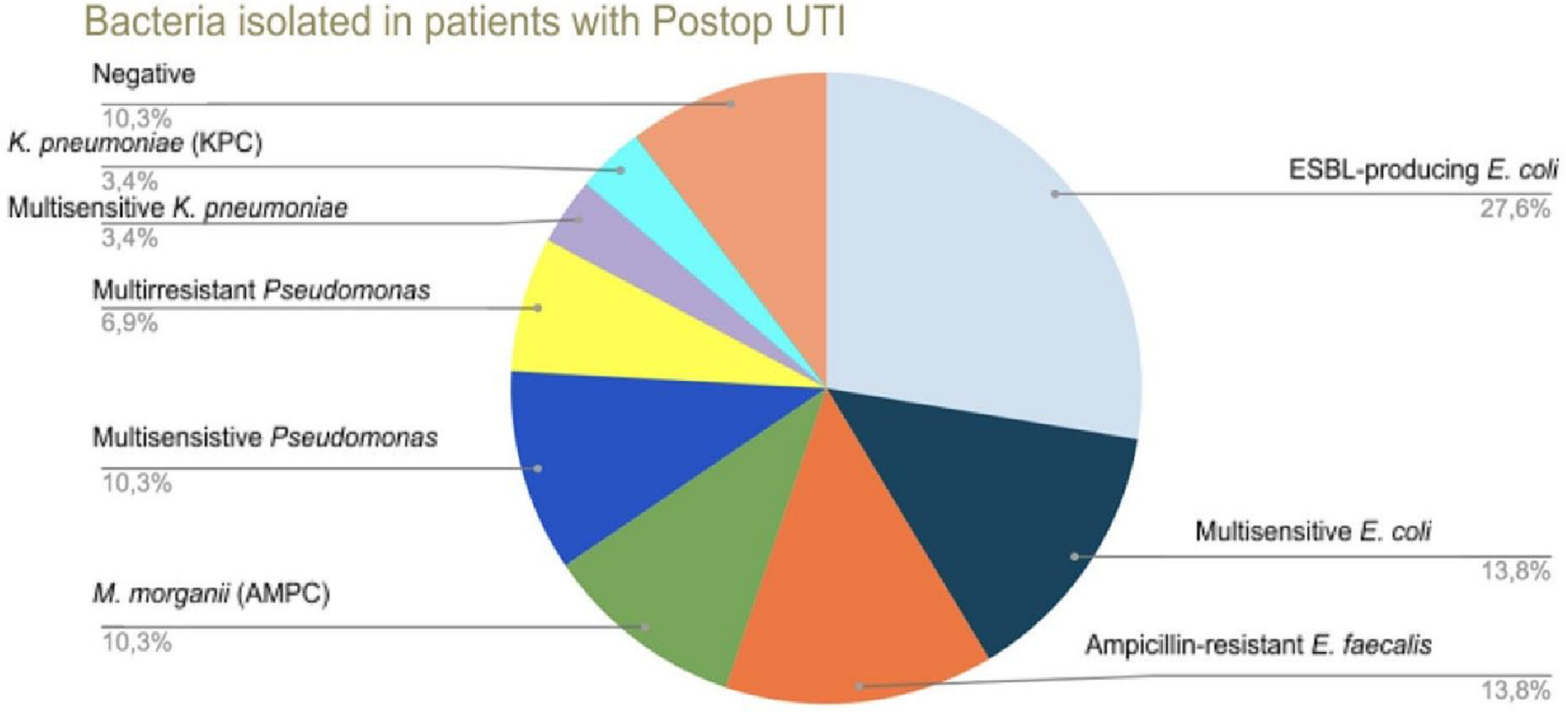
Isolated bacteria in the urine culture of the infectious complication in the two cohorts.

Infectious complications occurred in 7.2% of patients, 4% with lower UTI, 1.8% with upper UTI and 1.4% with urinary sepsis. Isolation was not achieved in the UC in 10.3% of the infected patients, and all patients completed an empiric management scheme with broad‐spectrum antibiotics. Multidrug‐resistant bacteria were isolated in 69% of infected patients, being higher in the group with urinary infection and extended prophylaxis with 94% versus 47.3% in the group of patients with urinary infection and standard prophylaxis. Figure 1 shows the distribution of bacteria isolated in patients with postoperative UTI. These patients had an early microbiological input; however, the first management was empirical with fourth generation cephalosporin (cefepime) and carbapenem (meropenem) following institutional guidelines, followed by antibiogram susceptibility pattern treatment.

The microbiological data did not mirror the preoperative UC because we only analysed patients with negative UC previous to surgical procedure.

Patients had not documented UTI previous to surgical procedure, confirmed with negative UC, and the antibiotic previous to surgery was prophylaxis following the institutional protocols.

The frequency of postoperative urinary infection was significantly higher in the extended prophylaxis group with 11.7% versus 5.1% in the standard prophylaxis group (RR = 2.32; 95% CI: 1.24–4.34, *p* = 0.007); however, most patients developed lower UTI, occurring in 6.8% of patients with extended prophylaxis and 2.7% of patients with standard prophylaxis (RR = 2.54; 95% CI: 1.07–6.00). In patients with upper UTI and sepsis, no statistically significant difference was found between the two cohorts. The risk of having multidrug‐resistant bacteria among the complications was 3.1 times higher in the extended prophylaxis group (RR 3.1; 95% CI: 1.33–7.59) (Table 3).

**TABLE 3:** Multivariate analysis model of the type of prophylaxis for infectious complications, controlled for confounding variables.

Complication	Extended prophylaxis *n* = 162	Standard prophylaxis *n* = 336	Bivariate		Multivariate	
			Risk estimation	*P* value	Risk estimation	*P* value
Post OP infection	19 (11.7%)	17 (5.1%)	RR 2.4; 95% CI (1.25–4.93)	0.007	RR 2.2; 95% CI (1.10–4.48)	0.026
Low post OP UTI	11 (6.8%)	9 (2.7%)	RR 2.5; 95% CI (1.07–5.99)	0.029	RR 2.8; 95% CI (1.10–7.37)	0.030
Upper post OP UTI	5 (3.1%)	4 (1.2%)	RR 2.5; 95% CI (0.70–9.52)	0.137	RR 1.8; 95% CI (0.42–7.83)	0.420
Sepsis	3 (1.9%)	4 (1.2%)	RR 1.5; 95% CI (0.35–6.86)	0.55	RR 1.6; 95% CI (0.36–7.74)	0.510
Multidrug‐resistant bacteria	9 (9.9%)	16 (2.7%)	RR 3.6; 95% CI (1.66–8.16)	0.001	RR 3.1; 95% CI (1.33–7.59)	0.009

## 4 DISCUSSION

Urolithiasis is a pathology with a significant incidence and prevalence in developed countries.^15^ The treatment of non‐expulsable calculi is very diverse.^1^, ^16^ The choice depends essentially on the number, size, composition and location of the stones.^1^, ^16^, ^17^, ^18^ The incidence of postoperative urinary infection is reported in 0.2–15% and urinary sepsis in 0.1–4.3%.^19^ The most frequently isolated bacterium is *E. coli*, followed by *Proteus* and *Pseudomonas*, and it is noteworthy that in up to 55.8% of cases, the postoperative UC is reported as negative.^9^ We found similar outcomes in our study, with urinary infection documented in 7.2% of the patients, upper UTI in 1.8% and urinary sepsis in 1.4% of patients. In this series, the most frequently isolated bacterium corresponds to extended‐spectrum beta‐lactamase (ESBL)‐producing *E. coli* (27.6%), and negative UC was reported in 10.3% of patients. This high rate of multidrug‐resistant infection can be explained by the fact that in Colombia there are many factors related to antibiotic misuse as self‐medication, inadequate patient compliance in antibiotic regimes, use of antibiotics as a treatment for non‐bacterial infections diseases, limited training and education in antibiotic use, and the easy access to antibiotics.^20^ Besides, health professionals and students have limited training and education in antibiotics that could contribute to an increasing of bacterial resistance.^20^

It has been described that AP decreases the incidence of pyuria (RR = 0.65; 95% CI: 0.51–0.82; *p* = 0.0005) and bacteriuria (RR = 0.26; 95% CI: 0.12–0.60) with a statistically significant difference.^10^ The rates of bacteriuria and febrile UTI are lower in patients receiving preoperative antibiotics 4.5% versus 11.8% (*p* = 0.09) and 1.3% versus 5.9% (*p* = 0.09), respectively.^15^ In this study, all patients received prophylaxis with infection rates consistent with these reports, and the risk of lower UTI was higher in the extended prophylaxis group compared with standard prophylaxis, 6.8% versus 2.7% (RR = 2.8; 95% CI: 1.10–7.35, *p* = 0.030).

Gravas et al.^21^ evaluated the efficacy of AP on postoperative infection rate in patients with negative UC that underwent ureteroscopy in 2010, finding a higher risk of postoperative lower UTI in the group of patients without AP with an OR of 1.27, 95% CI (0.40–4.00). Also described a low prevalence of fever (2.2%) with an OR of 1.84, 95% CI (0.69–4.94) in patients undergoing stone removal, although without a statistically significant difference.^21^ In our study, extended prophylaxis showed no statistical difference in the development of upper UTI (RR = 1.8; 95% CI: 0.42–7.83) or sepsis RR = 1.6; 95% CI (0.36–7.74) *p* = 0.51.

Chew et al.^22^ evaluated whether prophylactic antibiotics reduce UTIs after ureteroscopy for stone treatment, comparable with our study. All patients received a single dose of antibiotic prior to stone treatment, and a subset of patients also received postoperative antibiotics. A total of 9.9% of patients developed UTI, with no difference between patients who received a single dose compared with those who received more postoperative doses, *p* = 0.1457. Associated risk factors were preoperative stent placement, nephrostomy and urethral catheter.^22^ In this study, the risk of postoperative urinary infection was significantly higher in the extended prophylaxis group (11.7% vs. 5.1%; RR = 2.2; 95% CI: 1.10–4.48), and the multivariate analysis was controlled by age, use of ureteral access sheath, hydronephrosis and pre‐surgical diversion.

Kim et al.^23^ evaluated febrile UTIs after ureteroscopy for stones treatment, reporting that 14.1% of patients developed febrile UTI, and 44.2% had positive postoperative culture, in which 25.6% of them had isolation of *P. aeruginosa*. Only one patient presented other bacteria isolated in the postoperative culture and corresponded to *E. coli*, *P. mirabilis*, *Enterococcus faecalis*, *Enterobacter cloacae*, *Citrobacter amalonaticus* and *Achromobacter xylosoxidans*.^23^ Another study conducted in Korea found that the most frequently isolated bacteria in infectious complications correspond to ESBL‐producing *E. coli* evidenced in 17.6% of patients with febrile postoperative infection after ureteroscopy, followed by multisensitive *E. coli*, *P. aeruginosa* and *Acinetobacter baumannii*, each one with a frequency of 11.1%.^24^ This is consistent with the isolations in our sample, where ESBL‐producing *E. coli* was the most frequent bacterium, isolated in 27.6% of patients, followed by multisensitive *E. coli and E. faecalis*, each accounting for 13.8%. Multisensitive and multidrug‐resistant *Pseudomonas* occurred in 10.3% and 6.9% of patients, respectively.

There is a growing resistance to antibiotics that can cause the failure of therapeutic regimes.^7^, ^9^ In our sample, 69% of infected patients had multidrug‐resistant bacteria: ESBL‐producing *E. coli* was isolated in 27.6%, ampicillin‐resistant *E. faecalis* in 13.8%, *Morganella morganii* (AMPC) in 10.3%, multidrug‐resistant *Pseudomonas* in 6.9% *and Klebsiella pneumoniae* (KPC) in 3.4%. The risk of multidrug‐resistant infections was higher in the extended prophylaxis group (RR = 3.1; 95% CI: 1.33–7.59; *p* = 0.009), which emphasises the importance of evaluating the risk–benefit of extended prophylaxis compared with the standard.

AP in patients with negative UCs who undergo fURS varies according to the protocol of each institution. A total of 162 patients with extended prophylaxis and 336 with standard prophylaxis were included; the two groups were comparable in terms of preoperative demographic and clinical variables, except for gender, stone size, preoperative diversion and preoperative hydronephrosis. There was a greater number of male patients in the extended prophylaxis group with 60% versus 49% in the standard prophylaxis group. The presence of preoperative hydronephrosis and urinary diversion was higher in the standard prophylaxis group.

The incidence of complications was 10.3%, of which 7.2% were infection‐related complications. The risk of UTI was significantly higher in the extended prophylaxis group with a RR of 2.2 (95% CI: 1.10–4.48), without affecting its result in the multivariate study when including variables such as age, the use of ureteral access sheath in surgical procedure, urinary diversion and pre‐surgical hydronephrosis. The risk of upper UTI and sepsis did not show a statistical difference between the two types of prophylaxis.

The risk of developing infections due to multidrug‐resistant bacteria is greater in patients with endourology interventions.^13^ In patients who developed UTI, 69% had UC with multidrug‐resistant bacteria, with ESBL‐producing *E. coli* being the most frequent, which occurred in 27.6% of patients. Regarding the risk of infection by multidrug‐resistant bacteria, it occurred more frequently in the extended prophylaxis cohort with 9.9% versus 2.7% in the extended prophylaxis cohort, with the risk being 3 times higher in the extended prophylaxis cohort with a statistically significant difference.

The main limitation of our study is the retrospective design, which implies observation and selection bias; however, to maintain the validity of the study, the two cohorts and the infectious outcomes in terms of clinical and paraclinical diagnosis were clearly defined. In addition, a multivariate study was carried out with the confounding variables, and no difference was found in the impact of the outcomes. Patient follow‐up is another limitation, given that some patients with infectious complications were able to consult other health centres, which impacts the results.

## 5 CONCLUSIONS

In patients with negative UC who underwent fURS in a fourth‐level hospital, complications occurred in one out of 10 patients. Overall, 7.2% presented postoperative infection: 11.7% of patients with extended prophylaxis and 5.1% of patients with standard prophylaxis. The risk of UTI was 2.2 times higher in patients with extended prophylaxis, without demonstrating an impact on the incidence of upper UTI or sepsis. Variables such as age, use of ureteral access sheath, preoperative diversion and hydronephrosis did not affect the outcome. Overall, 1.4% of patients developed sepsis of urinary origin, without finding differences between the groups. The isolation of multidrug‐resistant bacteria occurred in 69% of patients with postoperative infection, and the most frequent bacteria were ESBL‐producing *E. coli*. The risk of multidrug‐resistant bacteria is 3 times higher in the extended prophylaxis group. Therefore, standard prophylaxis 60 min before the surgical procedure offers a lower risk of UTI and reduces the risk of isolation of multidrug‐resistant bacteria.

## AUTHOR CONTRIBUTIONS

The authors confirm contribution to the article as follows: Conceptualisation and methodology: DMMG, CBC, AFP, DAC, DCN. Data collection: DMMG, MAC, JCAR, MABC, NPB. Software and data curation: DMMG, MIP, JDV. Writing: DMMG, CBC, AFP, DCN, MCMM.

## CONFLICT OF INTEREST STATEMENT

No conflicts of interest nor financial conflicts to declare.

## References

[1] Skolarikos A , Neisius A , Petrik A , Somani B , Thomas K , G. Gambaro , et al. EAU Guidelines on Urolithiasis. 2022.

[2] Scales CD , Krupski TL , Curtis LH , Matlaga B , Lotan Y , Pearle MS , et al. Practice variation in the surgical management of urinary lithiasis. J Urol. 2011;186(1):146–50. 10.1016/j.juro.2011.03.018 21575964 PMC3280611

[3] Ivan S , Sindhwani P . Comparison of guideline recommendations for antimicrobial prophylaxis in urologic procedures: variability, lack of consensus, and contradictions. Int Urol Nephrol. 2018;50(11):1923–37. 10.1007/s11255-018-1971-1 30145652

[4] Díaz Pérez D , Laso García I , Sánchez Guerrero C , Fernández Alcalde Á , Ruiz Hernández M , Brasero Burgos J , et al. Sepsis urinaria tras tratamiento endourológico de la litiasis por ureterorrenoscopia. Actas Urol Esp. 2019;43(6):293–9. 10.1016/j.acuro.2019.02.001 31056221

[5] Mitsuzuka K , Nakano O , Takahashi N , Satoh M . Identification of factors associated with postoperative febrile urinary tract infection after ureteroscopy for urinary stones. Urolithiasis. 2016;44(3):257–62. 10.1007/s00240-015-0816-y 26321205

[6] Wan D , Sun S , Kim W . Risk factors of infectious complication after ureteroscopic procedures of the upper urinary tract. 2013. 10.1007/s10156-013-0632-7 23783396

[7] Ramaswamy K , Shah O . Antibiotic prophylaxis after uncomplicated ureteroscopic stone treatment: is there a difference? J Endourol. 2012;26(2):122–5. 10.1089/end.2011.0360 22003847

[8] Rané A , Cahill D , Saleemi A , Montgomery B , Palfrey E . The issue of prophylactic antibiotics prior to flexible cystoscopy. Eur Urol. 2001;39(2):212–4. 10.1159/000052438 11223682

[9] Concia E , Azzini A . Aetiology and antibiotic resistance issues regarding urological procedures. J Chemother. 2014;26(sup1):S14–23. 10.1179/1120009X14Z.000000000233 25245707

[10] Lo CW , Yang SS , Hsieh CH , Chang SS . Effectiveness of prophylactic antibiotics against post‐ureteroscopic lithotripsy infections: systematic review and meta‐analysis. Surg Infect (Larchmt). 2015;16(4):415–20. 10.1089/sur.2014.013 26207401

[11] Marino Sabo E , Stern J . Approach to antimicrobial prophylaxis for urology procedures in the era of increasing fluoroquinolone resistance. Ann Pharmacother. 2014;48(3):380–6. 10.1177/1060028013517661 24396088

[12] Lightner DJ , Wymer K , Sanchez J , Kavoussi L . Best practice statement on urologic procedures and antimicrobial prophylaxis. J Urol. 2020;203(2):351–6. 10.1097/JU.0000000000000509 31441676

[13] Greene DJ , Gill BC , Hinck B , Nyame YA , Almassi N , Krishnamurthi V , et al. American urological association antibiotic best practice statement and ureteroscopy: does antibiotic stewardship help? J Endourol. 2018;32(4):283–8. 10.1089/end.2017.0796 29179565

[14] Leaper D , Burman‐Roy S , Palanca A , Cullen K , Worster D , Gautam‐Aitken E , et al. Prevention and treatment of surgical site infection: summary of NICE guidance. BMJ. 2008;337:1924. 10.1136/bmj.a1924 18957455

[15] Hsieh CH , Yang SS , Lin CD , Chang SJ . Are prophylactic antibiotics necessary in patients with preoperative sterile urine undergoing ureterorenoscopic lithotripsy? BJU Int. 2014;113(2):275–80. 10.1111/bju.12502 24127851

[16] Wein AJ , Kavoussi LR , Novick AC , Partin AW , Peters CA . Campbell‐Walsh Urology 11th ed.; 2016.

[17] Sola Galarza I , Martínez Ballesteros C , Alba DV , Carballido Rodríguez JA . Litiasis urinaria. Medicine (Baltimore). 2011;10(83):5601–11. 10.1016/S0304-5412(11)70146-9

[18] Ferrer Moret S , Bellerino Serrano E , Pérez MD . Litiasis renal: criterios de estudio, derivación y tratamiento. FMC. 2015;22:301–11.

[19] De CV , Keller EX , Somani B , Giusti G , Proietti S , Rodriguez M , et al. Complications of ureteroscopy: a complete overview. World J Urol. 2019;192(5):1–20. 10.1007/s00345-019-03012-1 31748953

[20] Toro‐Alzate LF . Antimicrobial Resistance in Colombia under the scope of One Health approach [thesis]. 2020.

[21] Gravas S , Etemadian M , Unsal A , Barusso G , Addessi AD , Krambeck A , et al. Postoperative infection rates in patients with a negative baseline urine culture undergoing ureteroscopic stone removal: a matched case‐control analysis on antibiotic prophylaxis from the CROES URS global study. J Endourol. 2015;29(2):171–80. 10.1089/end.2014.0470 25072350

[22] Chew BH , Flannigan R , Kurtz M , Gershman B , Arsovska O , Paterson RF , et al. A single dose of intraoperative antibiotics is sufficient to prevent urinary tract infection during ureteroscopy. J Endourol. 2016;30(1):63–8. 10.1089/end.2015.0511 26413885

[23] Kim JW , Lee YJ , Chung JW , Ha YS , Lee JN , Yoo ES , et al. Clinical characteristics of postoperative febrile urinary tract infections after ureteroscopic lithotripsy. Investig Clin Urol. 2018;59(5):335–41. 10.4111/icu.2018.59.5.335 PMC6121018 30182079

[24] Kim DS , Yoo KH , Jeon SH , Lee SH . Risk factors of febrile urinary tract infections following retrograde intrarenal surgery for renal stones. Medicine (Baltimore). 2021;100:e25182.1–4. 33787599 10.1097/MD.0000000000025182 PMC8021282

